# Quitting on TikTok: Effects of Message Themes, Frames, and Sources on Engagement with Vaping Cessation Videos

**DOI:** 10.1080/10810730.2024.2394774

**Published:** 2024-08-26

**Authors:** JIAXI WU, JESSICA L. FETTERMAN, JENNIFER CORNACCHIONE ROSS, TRACI HONG

**Affiliations:** 1 Annenberg School for Communication, University of Pennsylvania, Philadelphia, PA, USA; 2 Evans Department of Medicine and Whitaker Cardiovascular Institute, Boston University Chobanian & Avedisian School of Medicine, Boston, MA, USA; 3 Department of Health Law, Policy & Management, Boston University School of Public Health, Boston, MA, USA; 4 College of Communication, Boston University, Boston, MA, USA

## Abstract

This study examined how message themes, frames, and sources in vaping cessation videos on TikTok influenced positive (i.e. likes, shares, positive comments regarding quitting vaping) and negative video engagement (i.e. negative comments regarding quitting vaping). TikTok videos (*N* = 412) with the hashtags #quitvaping and #quittingvaping were analyzed. Aspect-based sentiment analysis was conducted to evaluate the sentiment of quitting vaping in comments. Negative binomial regression models predicted video engagement from six message themes, ratios of gain and loss frames, and message sources. Themes related to nicotine addiction and physical health effectively drove positive engagement, such as likes and shares. The theme of harmful chemicals elicited mixed responses, generating both positive and negative comments regarding quitting vaping. Videos with a higher ratio of gain frames led to more positive engagement, including likes, shares, and positive comments regarding quitting vaping. Sources with informal expertise (e.g. those who have successfully quit vaping) and current e-cigarette users were more effective in engaging the TikTok audience than non-expert and non-user sources. These findings provide insights into messaging strategies that can effectively engage TikTok audiences and encourage vaping cessation.

Approximately 2.13 million US youth reported using e-cigarettes, commonly known as vaping, in the past 30 days in 2023 (Birdsey et al., 2023). Despite the popularity of e-cigarette use among youth, 53.8% of youth under 18 years of age who currently vape express a desire to quit (Cuccia et al., 2021). However, vaping cessation poses challenges due to factors including a higher addictive potential compared to combustible cigarettes (Jankowski et al., 2019), the diversity of vaping products, ease of use, and the social acceptance of vaping among peers (Sanchez et al., 2021). As a result, over 2 in 3 youth who currently vape reported failed attempts to quit vaping in the past year (Dai et al., 2023).

Previous research has demonstrated the effectiveness of smoking cessation campaigns in promoting quitting behaviors among youth and young adult smokers (Klein et al., 2005; Murphy-Hoefer et al., 2020). However, limited campaign initiatives exist to encourage vaping cessation among youth currently using e-cigarettes (Wu et al., 2024). Most national vaping-related health campaigns, such as the FDA’s The Real Cost Youth E-cigarette Prevention Campaigns primarily focus on vaping prevention among youth rather than vaping cessation.

Using TikTok to reach youth about vaping cessation may be particularly effective due to its immense popularity among this demographic. In the US, 67% of youth aged 13–17 report having used TikTok, with 16% reporting almost constant use (Research Center, 2022). This research aims to provide insights into effective messaging strategies for promoting vaping cessation among young people by analyzing engagement with quitting vaping videos on TikTok. Theoretically, this research extends the framing and message source effects to the context of vaping cessation. Furthermore, by examining different types of social media engagement—such as likes, shares, positive, and negative comments regarding quitting vaping—this study provides valuable insights into using engagement metrics as proxies for assessing the effectiveness of social media health campaigns.

## Engaging Audiences with Vaping Cessation TikTok Videos

Health campaigns have increasingly utilized social media in recent decades to reach youth and young adults (Willoughby & Noar, 2022). Social media engagement is broadly defined as any action where users interact, share, and create content within their networks (McCay-Peet & Quan-Haase, 2016). In health campaigns using social media, engagement has also become commonplace in campaign evaluations, serving as a proxy for message effectiveness (Chan et al., 2020; Kite et al., 2019; Kornfield et al., 2015).

### Engagement as Part of Behavioral Change

The Integrated Behavioral Model posits that positive attitudes, perceived social norms, and personal agency regarding a behavior predict behavioral intentions, which subsequently influence actual behavior (Fishbein, 2009; J. Lee, 2017). People like social media posts for various reasons, such as socializing, giving feedback, sharing interests, and enjoyment; however, liking generally indicates a direct expression of positive sentiment (Dhir et al., 2018; Gan, 2017; J. Lee, 2017). Furthermore, individuals tend to share social media content that aligns with their beliefs (Aruguete & Calvo, 2018). Therefore, liking and sharing a post on social media may signal audience interest and positive attitudes toward the content, potentially serving as a “priming step” to behavior change (Chan et al., 2023; Kite et al., 2023). Based on the Integrated Behavioral Model, positive comments about promoted health behaviors suggest a favorable attitude toward adopting the behavior, whereas negative comments may reflect reluctance to embrace the recommended behavior.

### Engagement as Persuasive Cues

The bandwagon effect is when people conform to the behavior and attitudes of others due to the belief that such behavior and attitudes are popular, desirable, or socially acceptable (Cialdini & Goldstein, 2004; Cialdini et al., 1999). In the context of social media communication, bandwagon cues, such as a large number of likes, shares, and positive comments, can trigger the bandwagon effect by signaling popularity and social acceptance (Kim, 2018; Li & Sundar, 2022; Luo et al., 2020; Walther et al., 2010). For example, Luo et al. (2020) found that news headlines on Facebook with many likes were rated more credible than news with fewer likes. Health campaigns that received a greater number of positive comments were evaluated more favorably than those campaigns associated with more negative comments (Walther et al., 2010) and fewer positive comments (Li & Sundar, 2022). Moreover, high shares increased perceptions of message influence and preventive health behavioral intentions (Kim, 2018).

Therefore, engagement with social media health campaigns not only reflects how audiences respond to the post but also influences how the post is processed. This study focuses on metrics including positive engagement (i.e., likes, shares, positive comments about quitting vaping) and negative engagement (i.e., negative comments about quitting vaping) to identify effective features for future vaping cessation social media campaigns. Specifically, we focus on examining the effect of message source and content features, including message themes and frames on audience engagement with vaping cessation TikTok videos.

## The Effect of Message Themes on Video Engagement

Previous research has identified the following common themes in vaping-related health messages: 1) physical health outcomes (Boynton et al., 2022; Kong et al., 2016), 2) mental health outcomes (Grant et al., 2019), 3) harmful chemicals in vape products (Kresovich et al., 2022; Lazard, 2021; Popova et al., 2021), 4) nicotine addiction (Rohde et al., 2021), 5) the negative social image associated with vaping (Cavallo et al., 2019), and 6) financial costs of vaping (Kong et al., 2016).

Themes addressing nicotine addiction, harmful chemicals, and negative health outcomes led to higher perceived message effectiveness among youth (Boynton et al., 2022). Lazard (2021) found that themes related to physical health outcomes were perceived as the most effective, surpassing themes on chemicals in vapes, mental health outcomes, and nicotine addiction. Additionally, nicotine addiction themes were less effective in eliciting negative affect compared to physical health effects and chemicals in vapes (Noar et al., 2019; Rohde et al., 2022)

Notably, these theme-based studies pertained to vaping prevention instead of vaping cessation. The current study explores what message themes receive more engagement with TikTok vaping cessation videos. The following research question was proposed:

**RQ1:** What are the associations between the six pre-identified themes and both positive and negative engagement with vaping cessation TikTok videos?

## The Effect of Message Frames on Video Engagement

Health messages can be framed to emphasize either the benefits of a behavior (gain frame) or the consequences of not engaging in it (loss frame) (Rothman & Salovey, 1997). Studies suggest that loss-framed messages are more persuasive for detection behaviors like cancer screening, while gain-framed messages are more effective for promoting prevention behaviors such as exercise or quitting tobacco products (O’Keefe & Jensen, 2007, 2009; Rothman & Salovey, 1997). Research on gain and loss frames in the context of vaping prevention has yielded mixed results (Cai & Zhao, 2021; Calabro et al., 2019; Kong et al., 2016). However, no studies have specifically examined the effects of gain and loss frames on promoting vaping cessation (Wu et al., 2024). Despite the distinctions between cigarette cessation and vaping cessation concerning the products involved, a previous meta-analysis suggests that gain-framed messages were more likely than loss-framed messages to encourage smoking cessation (Gallagher & Updegraff, 2012).

### Ratio of Gain and Loss Frames

Previous experimental studies have predominantly focused on comparing pure gain-framed and loss-framed messages (O’Keefe & Jensen, 2009; O’Keefe & Nan, 2012). However, in real-life scenarios, the incorporation of both gain and loss frames in health messages, particularly within the context of TikTok videos, is common. The Emotions-as-Frames model (EFM, Nabi, 2003, Nabi, 2007) argues that loss-framed messages, emphasizing the negative consequences of not adopting recommended behaviors, tend to evoke negative emotions such as fear and guilt (Bilandzic et al., 2017; Seo et al., 2013; Wong et al., 2013). Conversely, gain-framed messages are more likely to elicit positive emotions such as hope (Bilandzic et al., 2017, Nabi et al., 2018). Furthermore, EFM suggests positive emotions enhance the persuasive impact of gain framing, while negative emotions strengthen the influence of loss framing (Nabi et al., 2019).

Increasing the ratio of gain to loss frames in a message could intensify emotional responses. Given the documented advantage of gain frames in smoking cessation literature, we posit the following hypotheses:

**H1**: Vaping cessation TikTok videos with a higher ratio of gain frames elicit more positive social media engagement and less negative engagement than videos with a lower ratio of gain frames.

**H2**: Vaping cessation TikTok videos with a higher ratio of loss frames elicit less positive social media engagement and more negative engagement than videos with a lower ratio of loss frames.

## The Effect of Message Sources on Video Engagement

A message source is the individual, group, or organization that the audience perceives as the communication originator (McGuire, 2013). The characteristics of a message source can contribute to attitudinal and behavioral change through two psychological processes: internalization and identification (El Hedhli et al., 2021; Kelman, 1961).

The internalization process can be manifested in terms of the expertise of message sources; formal experts, like healthcare professionals, can increase vaping risk perceptions among young adults (D. N. Lee & Stevens, 2022; Schmidt et al., 2016). In addition, recent research has acknowledged the persuasive effects of informal experts, which are individuals who have firsthand experience (i.e., experiential expertise) with specific health issues (Hocevar et al., 2017). In vaping cessation, individuals who have successfully quit possess informal expertise, drawing on their firsthand experiences and knowledge of the quitting process.

Identification is enhanced by source homophily, where similarities in beliefs, values, and social status between sender and recipient strengthen message impact (Rogers & Bhowmik, 1970; Z. Wang et al., 2008). Although the literature on youth preferences for vaping cessation sources is limited, research shows youth smokers prefer messages from peers who smoke (Latimer et al., 2012). When the recipient of a message perceives themselves to be relatable to the sender, the persuasive impact of the message tends to be stronger (Sohn & Choi, 2019). Thus, current e-cigarette users might be effective message sources for vaping cessation campaigns.

Given the inability to determine the vaping and quitting status of TikTok video viewers and the lack of research on different message sources in vaping cessation videos, we have developed the following research questions to evaluate the influence of various message sources on engagement.

**RQ2**: Do videos featuring formal experts, informal experts, and current e-cigarette users receive greater positive engagement and less negative engagement than videos that do not incorporate these message sources?

**RQ3**: Which of the message sources (formal experts, informal experts, current e-cigarette users) generate the highest positive engagement and the least negative engagement in vaping cessation TikTok videos?

## Methods

### Study Design and Data Collection

Using an open-source TikTok scraping tool (Nord, 2021), we collected all publicly available TikTok videos containing the hashtags #quitvaping and/or #quitvape posted between January 1^st^, 2022, and December 31^st^, 2022. In total, we collected 1,709 public TikTok videos, including associated metadata such as the number of video diggs (i.e., likes), comments, and follower counts. The comments associated with the 1,709 TikTok videos were collected, resulting in a total of 47,879 comments. We randomly sampled 50% of the 1,709 videos (*N* = 855) for the content analysis. The Institutional Review Board at a major university in the northeastern US exempted this study from review because it involved non-human subjects and used publicly available data.

### Sampling and Inclusion Criteria

**Figure 1.**
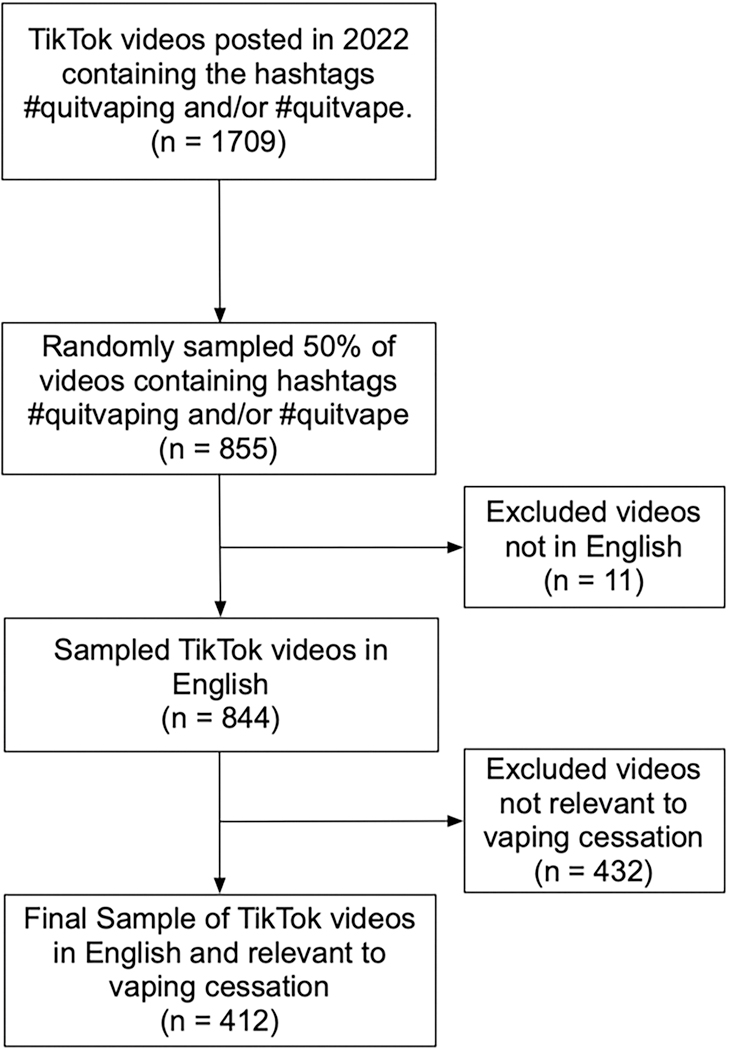
Video sampling procedure.

We first coded if the video was in English. Videos that were not in English were excluded from further analysis. Next, we determined the relevance of each video to vaping cessation. Only videos that explicitly mentioned quitting e-cigarettes were considered relevant to our study. For instance, videos that offered advice on quitting, shared personal experiences of quitting, or discussed the benefits of quitting were deemed relevant to quitting vaping. Figure 1 displays the sampling procedure used in this study.

### Intercoder Reliability

To attain high coding reliability, two coders were first trained on 50 videos that were not included in the sampled video dataset. Discrepancies were discussed to resolve coding disagreements in three separate meetings. Next, two coders independently coded 10% of the sample data (*N* = 86) for inter-coder reliability. Coding agreements were assessed with Cohen’s Kappa values, which were above 0.7 across all content variables, indicating a high level of intercoder reliability (Landis & Koch, 1977). The two trained coders then independently coded the rest of the videos. Table 1 displays the inter-coder reliability.

### Video Coding Features – Predictor Variables

The coding of message frames is contingent on message themes, as a frame can only be properly understood within the context of a specific theme. Therefore, we coded the presence/absence of six gain and/or loss-framed themes related to vaping from previous studies: 1) physical health outcomes; 2) mental health outcomes; 3) harmful chemicals in vape products; 4) nicotine addiction; 5) negative social image associated with and 6) financial costs of vaping. A video could contain both gain and loss-framed messages across six specific themes. Thus, a total of 12 gain/loss-framed themes were coded for each video.

#### Presence of Six Message Themes

The presence of each of the six themes was determined based on the inclusion of gain or loss-framed messages related to the coded theme.

#### Ratio of Gain Frames

We calculated the ratio of gain frames by dividing the number of gain-framed themes by the total number of present gain/loss-framed themes.

#### Ratio of Loss Frames

Similarly, we calculated the ratio of loss frames by dividing the number of loss-framed themes by the total number of present gain or loss-framed themes.

#### Message Source

A message source was categorized as a formal expert source (i.e., healthcare professionals) if the main character in a video introduced themselves as a healthcare professional or wore medical professional attire (e.g., white coats, scrub tops). In addition, a message source was determined as an informal expert (i.e., individuals who have successfully quit vaping) if the main character in the video indicated they had successfully quit vaping. Lastly, a message source was classified as a current user message source if the s character disclosed current e-cigarette use. Videos that did not contain any of the above three message sources were categorized as having non-expert and non-user sources.

### Video Engagement - Outcome Variables

#### Numbers of Likes and Shares

The number of likes and shares a video received was obtained during the scraping of the videos.

##### Positive and Negative Comments About Quitting Vaping.

To evaluate the sentiment of comments about quitting vaping, we conducted aspect-based sentiment analysis (ABSA) on all videos with at least one comment. In ABSA, “aspects” are attributes or components discussed in the text. We analyzed 47,879 comments using ABSApp (Pereg et al., 2019), identifying 152 initial aspects. ABSApp provided examples of text strings for each aspect, which guided us in manually selecting six relevant terms for quitting vaping: quit, journey, choice, quitting, decisions, and decision. We excluded irrelevant aspects such as years, anyone, dude, dreams, and kids.

We calculated aspect-based sentiment for each comment using an off-the-shelf LSA_*T*_-DeBERTa model. LSA_*T*_-DeBERTa demonstrates state-of-the-art performance across various natural language processing tasks by effectively capturing contextual information and semantic relationships within the text. The model achieves a macro-average performance score of 85% on multiple public datasets (Yang & Li, 2022). The model provided probabilities for negative, neutral, and positive sentiments. For instance, “Nicotine has nothing to do with our anxiety, I quit back in February and I’m just as anxious and depressed as I was before” was categorized as negative to quitting vaping, “How did you quit?” as neutral, and “I want to quit so badly, not sure why I keep putting it off” as positive. Comments were assigned to the sentiment category with the highest probability. We then summed the number of positive and negative comments about quitting vaping for each video with at least one relevant comment. We validated the model’s predictions on aspect sentiment regarding quitting vaping by manually coding 15% of the examined comments. The validation metrics demonstrate good performance, with an accuracy of 81.08%. Details of the validation process and results are provided in the supplementary materials.

### Statistical Analyses

Mixed-effect negative binomial models were utilized to test hypotheses and research questions, with each engagement metric (likes, positive and negative comments regarding quitting vaping, shares) treated as outcome variables respectively. The models included the following predictors: 1) the presence or absence of each of the six message themes, 2) a four-level categorical variable indicating the type of message source, and 3) a continuous variable representing the ratio of gain/loss frames in the video. To avoid multicollinearity, the ratio of gain frames and loss frames was entered separately as predictors, along with two other predictor variables in each of the negative binomial models.

The analyses were conducted using R (Version 1.4.1106) and the R package glmmADMB. All models included random effects of TikTok users and were adjusted for variables that could affect video engagement, including TikTok account follower counts (per thousand), video length (in seconds), and the total numbers of gain and loss-framed themes in the video. Videos featuring at least one of the six identified themes were included in the negative binomial analysis of likes and shares. Additionally, videos that mentioned at least one theme and received at least one comment were analyzed for positive and negative comments about quitting vaping.

## Results

### Descriptive Analysis Results

The 412 videos received over 83 million views on TikTok, with an average of 203,201 views per video (*SD* = 677,793). Videos received a mean of 248 comments (SD = 924, Mdn = 28, IQR = 89), 21185 likes (SD = 72,775, Mdn = 1,408, IQR = 5,119), and 368 shares (SD = 1,541, Mdn = 11, IQR = 76). The mean number of positive comments about quitting was 3 (SD = 7, Mdn = 1, IQR = 3), and the mean number of negative comments about quitting was 3 (SD = 7, Mdn = 1, IQR = 4).

#### Message Themes and Frames

Table 2 presents the presence of twelve gain- and loss-framed themes in English-language vaping cessation videos (*N* = 412). The most common theme was nicotine addiction, followed by physical health, mental health, harmful chemicals in vapes, financial impacts of vaping, and negative social perceptions of vaping. Exploratory inductive coding of the 135 videos without these six themes revealed that 56 (41%) featured individuals discussing their decision to quit vaping (see supplemental materials). Table 3 provides examples of gain and loss-framed messages for each theme. Among the 277 videos containing at least one of the identified themes, the average ratio of gain frames was 0.29 (SD = 0.37), while the ratio of loss frames was 0.71 (SD = 0.37).

#### Message Sources

Among the coded videos, 10 (2.4%) videos featured formal experts. Additional string-matching analyses using keywords like “doctor,” and “MD” did not find additional formal expert videos (supplementary materials). Furthermore, 54 (13.1%) videos showed informal experts, who indicated that they have successfully quit vaping, while 241 (58.5%) videos portrayed current e-cigarette user sources. Lastly, 107 (26.0%) videos included non-expert and non-user sources.

### Predicting Video Engagement with Message Themes, Frames, and Sources

Table 4 displays the results of mixed-effect negative binomial regression models.

#### Effects of Six Message Themes on Video Engagement

RQ1 examined the effects of six distinct message themes on video engagement. Negative binomial regression results revealed that the presence of the chemical theme was associated with both more negative (IRR = 2.74, *p* = .02, 95% CI = 1.15, 6.52) and positive comments (IRR = 2.15, *p* = .05, 95% CI = 1.01, 4.56) about quitting vaping. Additionally, the physical health theme was linked to more likes (IRR = 3.30, *p* = .01, 95% CI = 1.39, 7.86) and shares (IRR = 5.11, *p* = .003, 95% CI = 1.74, 15.05), while the addiction theme received more likes (IRR = 2.76, *p* = .05, 95% CI = 1.01, 7.50).

#### Effects of Gain and Loss Frames on Video Engagement

H1 proposed that a higher ratio of gain frames to the total number of gain and loss frames in a video would predict increased positive engagement and reduced negative engagement. The results suggest that videos with a higher ratio of gain frames elicited more likes (IRR = 2.79, *p* = .01, 95% CI = 1.23, 6.30), positive comments about quitting vaping (IRR = 1.86, *p* = .04, 95% CI = 1.04, 3.33), and more shares (IRR = 3.51, *p* = .01, 95% CI = 1.35, 9.12). However, no significant association was found between negative comments and the ratio of gain frames (IRR = 0.32, *p* = 1.40, 95% CI = 0.72, 2.72). Therefore, H1 was partially supported.

H2 proposed that a higher ratio of loss frames in a video would predict decreased positive engagement and increased negative engagement. The results suggest that videos with a higher ratio of loss frames elicited fewer likes (IRR = 0.36, *p* = .01, 95% CI = 0.16, 0.81), fewer positive comments about quitting vaping (IRR = 0.54, *p* = .04, 95% CI = 0.30, 0.96), and fewer shares (IRR = 0.28, *p* = .01, 95% CI = 0.11, 0.74). Additionally, no significant association was found between negative comments and the ratio of loss frames (IRR = 0.71, *p* = .32, 95% CI = 0.37, 1.38). Therefore, H2 was partially supported.

#### Effects of Message Sources on Video Engagement

RQ2 investigated that if TikTok vaping cessation videos featuring formal experts (i.e., healthcare professionals), informal experts (i.e., individuals who have successfully quit vaping), and current user sources (i.e., individuals who currently use e-cigarettes) generate more positive engagement and less negative engagement compared to videos featuring non-expert and non-user sources. Findings from negative binomial regressions showed that non-expert and non-user sources received fewer likes (IRR = 0.45, *p* = .04, 95% CI = 0.21, 0.97) than current user sources. In addition, non-expert and non-user videos were associated with more negative comments about quitting vaping than informal experts who have successfully quit vaping (IRR = 2.61, *p* = .03, 95% CI = 1.12, 6.07).

RQ3 asked which of the three message sources (formal experts, informal experts, current user sources) generate the highest engagement compared to one another. The results indicated that informal expert sources received both fewer positive comments (IRR = 0.40, *p* = .005, 95% CI = 0.21, 0.76) and fewer negative comments (IRR = 0.31, *p* = .002, 95% CI = 0.15, 0.64) about vaping than current user sources. No other significant differences were observed in video engagement when comparing the three types of message sources.

## Discussion

This study investigated how message themes, frames, and sources impact engagement with user-generated vaping cessation videos on TikTok. The primary themes in TikTok videos were physical health outcomes and nicotine addiction. On average, the videos featured a higher ratio of loss-framed messages over gain-framed messages. Additionally, over half of the videos featured individuals who disclosed current e-cigarette use, followed by non-expert non-user sources, informal experts who successfully quit, and formal experts such as doctors.

### Engagement with Vaping Cessation TikTok Videos

#### Themes and Video Engagement

Nicotine addiction emerged as the most prevalent theme, correlating with higher positive engagement (likes). Physical health, the second most common theme, also showed a positive correlation with positive engagement (likes and shares). Given that likes often indicate positive audience sentiment (Dhir et al., 2018; Gan, 2017; J. Lee, 2017), the increased correlation between likes and both nicotine addiction and physical health themes suggests potential effectiveness in future social media vaping cessation campaigns.

Sharing health-related information on social media can be driven by a desire to spread knowledge and show care for others (Iftikhar & Abaalkhail, 2017). Our findings suggest that people might regard physical health as significant enough to share within their networks. Future vaping cessation campaigns aim at increasing awareness and engagement with the issue of vaping cessation could emphasize the physical health effects of vaping.

Incorporating the theme of harmful chemicals in vaping products led to more positive comments about quitting, consistent with previous research on its effectiveness in prevention messages (Kreslake et al., 2022; Rohde et al., 2022). However, the theme of harmful chemicals also generated more negative comments about quitting. Previous research found that cigarette pack messages about toxic chemicals did not increase intentions to quit smoking, but increased awareness of chemicals and health harms (Brewer et al., 2019). Further research is needed to understand the effects of the chemical theme in vaping cessation and moderators that might affect the message effect.

#### Frames and Engagement

Aligning with the detection/prevention behavioral classification in gain and loss framing effects (Rothman & Salovey, 1997), our study found that a higher ratio of gain frames in vaping cessation videos was associated with increased likes, shares, and positive comments about quitting vaping. The benefits of incorporating gain frames may be explained by the heuristic processing of social media posts (Meinert et al., 2022). Individuals who rely on heuristic processing prefer positive information while avoiding negative information, consistent with the hedonic principle (Higgins, 1998). As the effectiveness of gain frames in persuasion depends on the intensity of positive emotions evoked (Bilandzic et al., 2017, Nabi et al., 2018), future TikTok vaping cessation campaigns may benefit from incorporating more gain-framed messages to maximize engagement (S. Wang et al., 2023). However, our results indicate that gain frames were not associated with reduced negative comments about quitting vaping compared to loss frames. Future research should explore why negative comments arise in response to social media health campaigns, considering factors like message reactance (Grandpre et al., 2003) and personal agency (Li & Sundar, 2022), to decrease negative engagement among audiences.

#### Sources and Engagement

When examining the effects of different message sources on video engagement, our study revealed an advantage in utilizing potentially relatable message sources who currently vape and informal expert sources. Vaping cessation videos featuring current users garnered more likes than those from non-expert, non-user sources. Additionally, videos featuring successful quitters received more positive comments compared to those featuring current users. Prior research has shown that “current teenaged smoker” and “successful teenaged quitter” were the top two preferred message sources for smoking cessation videos among youth (Latimer et al., 2012). Our study suggests that both current user and informal expert sources may effectively influence the audience’s attitudes toward quitting vaping.

Contrary to the hypothesis based on the internalization process of persuasion (Schmidt et al., 2016), our study found that formal expert sources such as doctors were not associated with more positive engagement. One possible explanation for the unexpected results could be the relatively small sample size of videos featuring formal expert sources (*N* = 10). Further research is needed to evaluate the effectiveness of including formal experts, like healthcare professionals, in vaping cessation TikTok videos.

#### Implications and Limitations of Using Engagement as Proxy Measures of Campaign Effectiveness

Drawing on the Integrated Behavioral Model (Fishbein, 2009; J. Lee, 2017) and the bandwagon effect (Cialdini & Goldstein, 2004; Cialdini et al., 1999), engagement metrics such as likes, shares, and comments may reflect audience perceptions of recommended behaviors, potentially precede behavioral change, and serve as persuasive cues in social media campaigns. For example, liking a brand on social media does not always result in purchasing the product (John et al., 2017). Therefore, while high engagement with health campaigns might signal positive sentiment, researchers have cautioned that such engagement does not always lead to meaningful attitude shifts or sustained behavior change (Thackeray et al., 2012). Moreover, engagement can also be influenced by factors unrelated to persuasion, such as entertainment value or peer influence (Chan et al., 2023). Research gaps include the aggregation of engagement types into a single score and a lack of focus on negative engagement, such as negative comments (Kite et al., 2023). Our study contributes to the literature by examining different engagement types and distinguishing positive and negative comments toward recommended health behaviors. However, a clearer theoretical understanding of the reasons and outcomes of engagement with social media health campaigns is still needed (Durkin et al., 2022; Fulgoni, 2016; Kite et al., 2019). Longitudinal and observational studies that link social media engagement to real-life health attitudes and behaviors could provide deeper insights.

Our study has limitations. Given our specific focus on TikTok vaping cessation videos, the findings may not apply to other social media platforms. Due to the content analysis nature, we lacked data on audience vaping status and age, preventing the examination of causal links between video exposure and quitting behaviors. Additionally, we were unable to study specific persuasive outcomes, nor did we analyze audience emotional responses to the videos. Moreover, it is essential to recognize that video engagement does not guarantee video persuasiveness.

Our study suggests that future TikTok vaping cessation campaigns could benefit from incorporating themes related to physical health, addiction, harmful chemicals, and gain-framed messages. Additionally, utilizing message sources current e-cigarette users and individuals who have successfully quit vaping, might enhance campaign engagement. The effectiveness of featuring formal experts, such as healthcare professionals, in vaping cessation TikTok videos warrants further research.

## Supplementary Material

Supplementary Material

## Figures and Tables

**Table 1.** Coding variables and intercoder reliability

Categories	Variable	Coding criteria	Coding categories	Cohen’s Kappa
Video relevance	Video language	The video is in English	1 = Yes 2 = No	1
	Video pertaining to vaping cessation	Videos that explicitly mentioned quitting e-cigarettes	1 = Yes 2 = No	0.93
Main character	Healthcare professionals: self-introduction	The main character in the video introduced him/her/them as a healthcare professional	1 = Yes 2 = No	0.77
	Healthcare professionals: medical outfit	The main character in the video wore an outfit for medical professionals such as scrubs or a white coat	1 = Yes 2 = No	0.98
	Individuals who currently vape	The main character in the video indicates that they currently use e-cigarettes	1 = Yes 2 = No	0.90
	Individuals who have successfully quit vaping	The main character in the video indicates that they successfully quit using e-cigarettes	1 = Yes 2 = No	0.89
Message frames and themes	Loss-framed physical health messages	If the video mentioned the negative consequences of vaping on physical health, such as lung disease and breathing problems	1 = Yes 2 = No	0.92
	Gain-framed physical health messages	If the video mentioned positive outcomes of quitting e-cigarettes on physical health, such as breathing deeper	1 = Yes 2 = No	0.91
	Loss-framed mental health messages	If the video mentioned the negative consequences of vaping on mental health, such as depression and increased stress	1 = Yes 2 = No	0.94
	Gain-framed mental health message	If the video mentioned positive outcomes of quitting e-cigarettes on mental health, such as feeling less stressed and less anxious	1 = Yes 2 = No	0.90
	Loss-framed harmful chemicals in vapes	If the video mentioned the negative consequences of vaping on inhaling unsafe components such as heavy metals	1 = Yes 2 = No	0.93
	Gain-framed harmful chemicals in vapes	If the video mentioned positive outcomes of quitting e-cigarettes in terms of not inhaling unsafe components in vapes	1 = Yes 2 = No	0.75
	Loss-framed nicotine addiction messages	If the video mentioned the negative consequences of vaping, such as addiction and feeling controlled by vaping	1 = Yes 2 = No	0.80
	Gain-framed nicotine addiction messages	If the video mentioned positive outcomes of quitting e-cigarettes in terms of feeling free from vaping	1 = Yes 2 = No	0.85
	Loss-framed negative social image messages	If the video mentioned the negative consequences of vaping on their social image, such as being identified as someone who vapes	1 = Yes 2 = No	0.95
	Gain-framed negative social image messages	If the video mentioned positive outcomes of quitting e-cigarettes in terms of feeling less pressure of being identified as someone who vapes	1 = Yes 2 = No	0.95
	Loss-framed financial costs messages	If the video mentioned the negative consequences of vaping on financial costs	1 = Yes 2 = No	0.98
	Gain-framed financial costs messages	If the video mentioned the positive outcomes of quitting e-cigarettes in terms of saving money	1 = Yes 2 = No	0.97

**Table 2.** Presence of themes and frames of the messages in TikTok cessation-related videos (*N* = 412)

Theme	Number of videos containing the theme	Number of videos containing gain-framed messages of the theme	Number of videos containing loss-framed messages of the theme
Nicotine addiction	199	76	174
Physical health	127	50	90
Mental health	86	35	61
Harmful chemicals in vaping products	46	5	11
Financial outcomes of vaping	39	23	19
Negative social images of vaping	12	2	11

**Table 3.** Examples of framed messages (paraphrased) related to coded themes in TikTok cessation-related videos

Theme	Gain-framed message	Loss-framed message
Physical health	Ditch your Puff Bars so your lungs last longer	After vaping for 7 years, I’ve been hospitalized, had the worst migraines, cold sweats . . . now I am quitting
Mental health	I am leaving vaping in 2022. It’s been two months without it, I feel great mentally, less anxious and happy	Reasons to quit: increased anxiety & depression
Nicotine addiction	Quitting helps me to escape from the cycle of nicotine addiction	Nicotine is addictive and can ruin your life
harmful chemicals in vapes	Point of me wanted to quit vaping is that there will be no harmful things in my lung anymore	Besides nicotine, e-cigarettes can contain harmful ingredients including led and tin
Financial outcomes of vaping	Saved $55 after day 4 of quitting	I literally have to buy vapes every day and it’s costing me a lot of money
Negative social images of vaping	I quit and now I am a better person.	Vaping is lame and people who vape are dumb

**Table 4.** Prediction of video engagement with message sources, themes, and ratios of gain and loss frames

	Likes (*N* = 277)		Negative comments regarding quitting vaping (*N* = 230)		Positive comments regarding quitting vaping (*N* = 230)		Shares (*N* = 277)	
	*P* value	IRR (CI)	*P* value	IRR (CI)	*P* value	IRR (CI)	*P* value	IRR (CI)
Follower count (per thousand)	**.01**	1.00(1.001–1.004)	.46	1.00(0.999–1.002)	.23	1.00(0.999–1.002)	**.01**	1.00(1.001–1.004)
Video length (in seconds)	.28	0.10(0.99–1.003)	.47	1.00(0.996–1.009)	.16	1.00(0.99–1.01)	.64	1.00(0.99–1.01)
Total number of themes	**.03**	0.55(0.32–0.93)	**.04**	0.61(0.38–0.98)	.43	0.85(0.56–1.28)	**.02**	0.48(0.26–0.88)
Source: formal expert (i.e., healthcare professional)	.0502	0.23(0.05, 1.002)	.86	0.89(0.24–3.29)	.38	0.60(0.19–1.90)	.60	0.64(0.12–3.52)
Source: informal expert (i.e., individuals successfully quit)	.16	0.54(0.23–1.28)	**.002**	0.31(0.15–0.64)	**.005**	0.40(0.21–0.76)	.84	0.91(0.35–2.37)
Source: non-expert and non-user sources	**.04**	0.45(0.21–0.97)	.50	0.80(0.43–1.51)	.12	0.63(0.35-–1.12)	.30	0.63(0.26–1.52)
Theme: Chemical	.15	1.92(0.78–4.72)	**.02**	2.74(1.15–6.52)	.**05**	2.15(1.01–4.56)	.09	2.53(0.87–7.36)
Theme: Physical health	**.01**	3.30(1.39–7.86)	.06	2.09(0.96–4.54)	.36	1.38(0.69–2.75)	**.003**	5.11(1.74–15.05)
Theme: Mental health	.57	1.26(0.57–2.81)	.11	1.74(0.88–3.45)	.75	1.10(0.61–2.00)	.09	2.26(0.89–5.70)
Theme: Addiction	**.05**	2.76(1.01–7.50)	.27	1.66(0.67–4.09)	.42	1.39(0.62–3.11)	.06	3.14(0.94-–10.51)
Theme: Financial	.74	1.19(0.43–3.26)	.57	1.31(0.51–3.35)	.53	0.75(0.31–1.83)	.83	0.88(0.25–3.03)
Theme: Social image	.97	0.96(0.18–5.04)	.99	1.01(0.19–5.52)	.82	1.21(0.23–6.30)	.59	1.73 (0.24–12.47)
Ratio of gain frames	**.01**	2.79(1.23–6.30)	.32	1.40(0.72–2.72)	**.04**	1.86(1.04–3.33)	**.01**	3.51(1.35–9.12)
Ratio of loss frames	**.01**	0.36(0.16–0.81)	.32	.71(0.37–1.38)	**.04**	.54(0.30–0.96)	**.01**	0.28(0.11–0.74)

The reference group for the message source is individuals who are currently using e-cigarettes. Each theme predictor was coded for the presence of either a gain or loss-framed theme. Reference group for message theme is the absence of the theme
