# Supplementary material for: Quitting on TikTok: Effects of Message Themes, Frames, and Sources on Engagement with Vaping Cessation Videos
Source: J Health Commun. Author manuscript; Available in PMC 2024 Nov 29. (PMC11606514; doi:10.1080/10810730.2024.2394774)

**Quitting on TikTok: Effects of Message Themes, Frames, and Sources on Engagement with Vaping Cessation Videos**

**Supplementary Material**

**Exploratory Coding of Videos Without Any of the Six Pre-identified Themes**

To comprehensively understand the content of all coded vaping-cessation-related TikTok videos without featuring one of the six pre-identified themes (n = 135) – namely, themes on nicotine addiction, physical health, mental health, harmful chemicals in vapes, financial outcomes of vaping, and negative social images of vaping – the first author conducted an exploratory inductive coding of the remaining 135 videos. Results from the exploratory coding indicate that the most prevalent theme was "decide to quit" (n = 56), featuring young individuals submerging their vapes into water with inspirational music to symbolize their commitment to quitting vaping. The second most frequent theme was quitting tips, such as eating gums and drinking water (n = 29), followed by updates on quitting progress (n = 15), quitting app/product ads (n = 12), jokes about quitting vaping but smoking cigarettes (n = 9), sharing the news of successfully quitting (n = 9), memes/jokes about quitting (n = 3), and telling people to quit without offering reasons (n = 2). Future studies should further explore how these unanalyzed vaping cessation videos may motivate other TikTok users to quit vaping, and how posts about one's decision to quit or updates on quitting progress may serve as facilitators for young people to quit vaping.

**String Matching to Identify Expert Account**

To identify potential expert accounts and associated videos from individuals who may not explicitly introduce themselves as healthcare professionals or wear medical attire (e.g., white coats, scrub tops), we conducted string matching using the following keywords in each account's handle and bio: dr, doc, doctor, MD, cardiologist, pulmonologist, physician, medicine, and pediatrician. However, no new expert accounts or videos were identified through this method. Additionally, we observed that the handles of all identified expert accounts contained at least one of the following keywords: dr, doc, and MD. These results suggest that medical professionals tend to convey their expertise on TikTok through identifiable signifiers when participating in vaping cessation discussions on TikTok.

**Model Performance and Validation**

To validate the model performance in predicting sentiments toward quitting vaping, 15% of the comments were randomly sampled for manual sentiment coding regarding quitting vaping. Positive sentiments were identified as comments expressing approval, encouragement, or enthusiasm about quitting vaping, such as “Quitting vaping was the best decision I ever made.” Neutral sentiments were those that did not convey strong feelings for or against quitting, typically providing information, asking questions, or making observations without clear approval or disapproval, for example, “I’m considering quitting but haven’t decided yet.” Negative sentiments expressed disapproval, doubt, or discouragement about quitting vaping, like “I tried quitting, but I felt terrible.” One team member coded the posts, while another team member reviewed the coding. Any discrepancies were discussed and resolved collaboratively.

Model validation metrics indicate that the model performs well overall, with an accuracy of 81.08%. It excels in identifying positive sentiments, achieving high precision (95.98%) and a strong F1 score (83.33%), although its recall (73.63%) could be improved, suggesting that some positive comments about quitting vaping are incorrectly classified as neutral or negative. For negative sentiments, the model shows high recall (94.59%) and a balanced F1 score (82.16%), but with lower precision (72.61%), suggesting some false positives where non-negative comments are incorrectly labeled as negative. The neutral sentiment classification shows moderate performance, with a precision of 72.30% and recall of 78.68%, leading to a lower F1 score (75.35%) compared to other classes (**Figure S1**).

**Figure S1. Confusion Matrix and Validation Metrics**


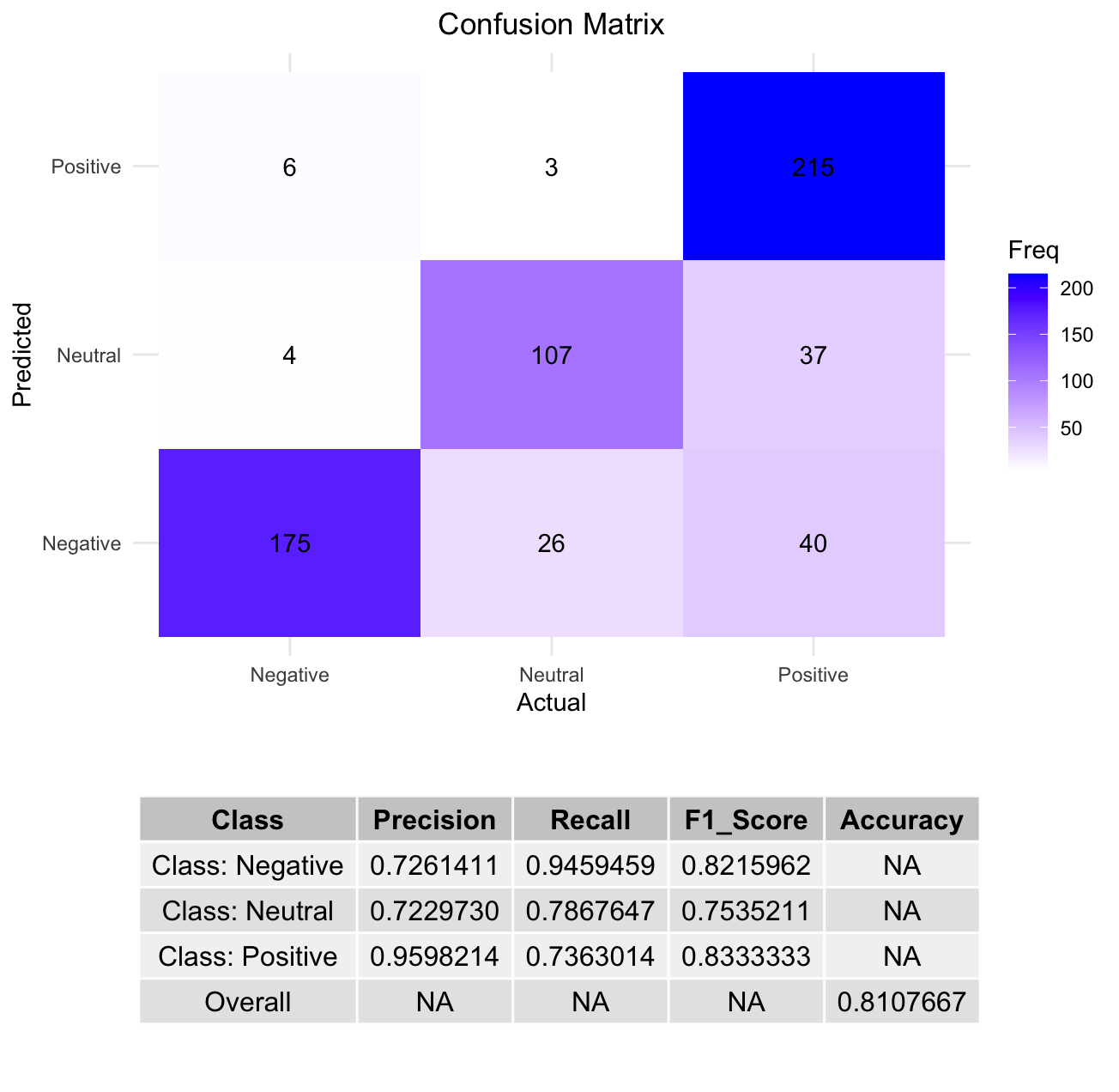

Supplement: Supplementary Material [file NIHMS2028146-supplement-Supplementary_Material.docx]
